# Cross-Species Analysis Reveals Co-Expressed Genes Regulating Antler Development in Cervidae

**DOI:** 10.3389/fgene.2022.878078

**Published:** 2022-05-18

**Authors:** Hengxing Ba, Min Chen, Chunyi Li

**Affiliations:** ^1^ Institute of Antler Science and Product Technology, Changchun Sci-Tech University, Changchun, China; ^2^ Jilin Provincial Key Laboratory of Deer Antler Biology, Changchun, China; ^3^ School of Life Sciences, Institute of Eco-Chongming (IEC), East China Normal University, Shanghai, China; ^4^ Yangtze Delta Estuarine Wetland Ecosystem Observation and Research Station, Ministry of Education & Shanghai Science and Technology Committee, Shanghai, China; ^5^ College of Chinese Medicinal Materials, Jilin Agricultural University, Changchun, China

**Keywords:** deer, antler-related genes, differential gene expression, antlerogenic periosteum, transcriptomics, Chinese water deer

## Abstract

Antlers constitute an interesting model for basic research in regenerative biology. Despite decades of being studied, much is still unknown about the genes related to antler development. Here, we utilized both the genome and antlerogenic periosteum (AP) transcriptome data of four deer species to reveal antler-related genes through cross-species comparative analysis. The results showed that the global gene expression pattern matches the status of antler phenotypes, supporting the fact that the genes expressed in the AP may be related to antler phenotypes. The upregulated genes of the AP in three-antlered deer showed evidence of co-expression, and their protein sequences were highly conserved. These genes were growth related and likely participated in antler development. In contrast, the upregulated genes in antler-less deer (Chinese water deer) were involved mainly in organismal death and growth failure, possibly related to the loss of antlers during evolution. Overall, this study demonstrates that the co-expressed genes in antlered deer may regulate antler development.

## Introduction

An ability to regenerate complex structures is widespread among lower organisms and is retained in some vertebrate species such as urodele amphibians. However, adult mammalian examples of epimorphic regeneration are extremely rare with the most dramatic example being the annual replacement of antlers in deer (Goss, 1983; Bubenik et al., 1990). Therefore, antlers constitute an interesting model for basic research in regenerative biology. On the other hand, despite decades of being studied, much is still unknown about the molecular regulation of antler development. Although being called cranial appendages, deer antlers do not grow directly from the head; instead, they generate and regenerate from the fully grown pedicles (antecedents of antlers). Pedicles and first antlers are both derived exclusively from the periosteum overlying the frontal crest of the deer head, known as the antlerogenic periosteum (AP) (Hartwig and Schrudde, 1974; Goss and Powel, 1985; Li and Suttie, 2000). Removal of the AP prior to pedicle initiation stops the pedicle and antler formation, and transplantation of the AP autologously to other sites of the body causes ectopic pedicle and antler to grow (Goss and Powel, 1985; Li et al., 2009a), thus enabling investigation at the molecular level. Each year during spring, fully calcified antlers are cast from the pedicles, which trigger the initiation of new antler regeneration from the pedicle stump (Li et al., 2002). In late spring and summer, antlers enter into the most rapid growth period (up to 2 cm/day) and are covered by a special type of skin, called velvet skin. In autumn, antlers are intensively calcified and shed the velvet skin to expose the bony antlers.

**FIGURE 1:**
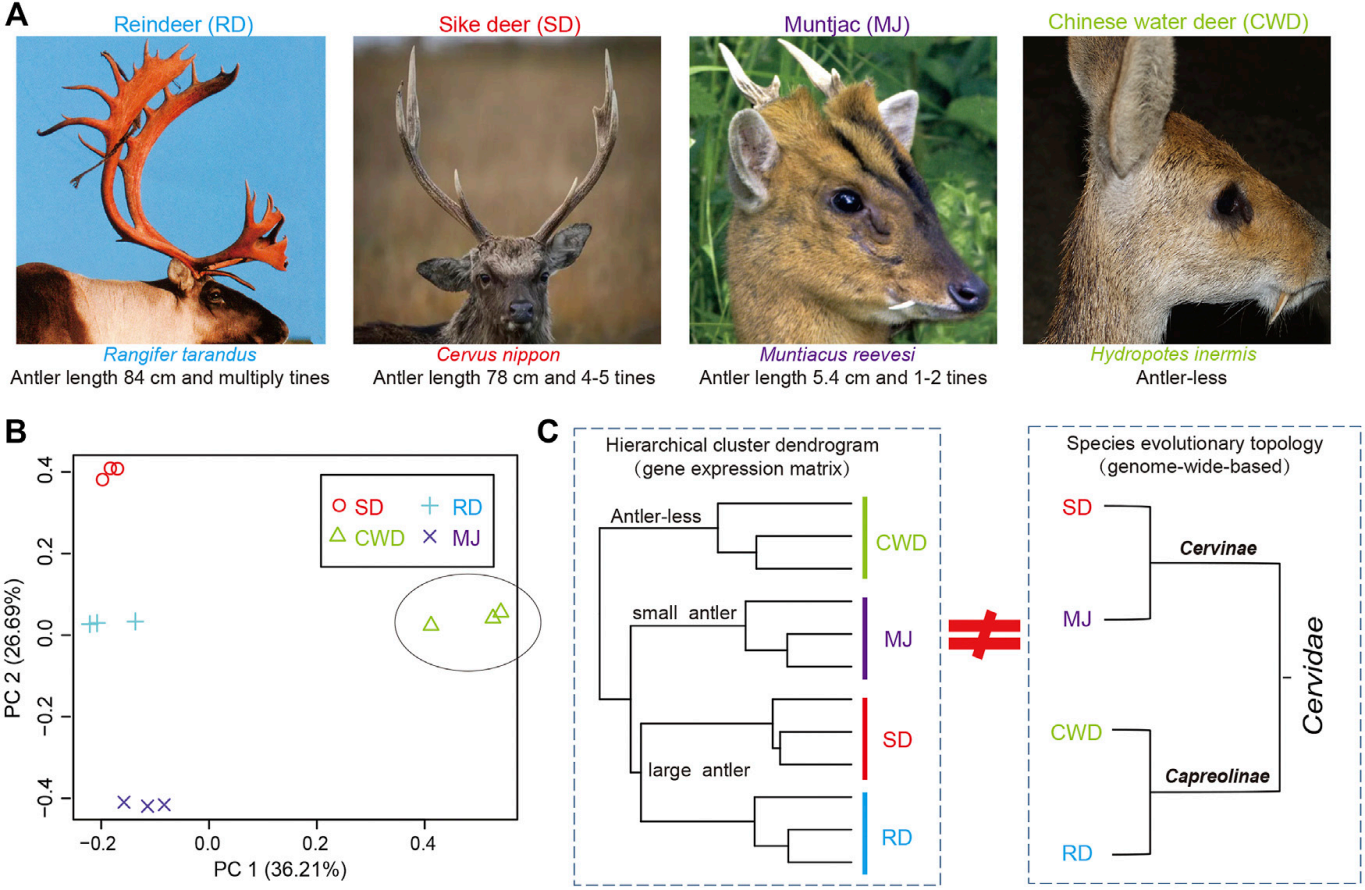
**(A)** Antlers of three-antlered deer (ATD) species including reindeer (RD), sika deer (SD), and muntjac (MJ); and one antler-less deer species, Chinese water deer (CWD). **(B)** Principal component analysis (PCA) of the gene expression of 12 samples across four deer species (three samples/species). **(C)** Left: hierarchical cluster analysis based on the gene expression matrix of 12 samples among four deer species; Right: evolutionary topology of four deer species obtained from the previous study (Chen et al., 2019).

Antlers exhibit polymorphism/polyphenism ranging from the large and complex structures grown by large species (Figure 1A) such as reindeer (*Rangifer tarandus*, antler length: ∼84 cm and multiple times) and sika deer (*Cervus nippon*, ∼78 cm and 3–5 times), to the small, simple antlers grown by small species such as the muntjac (Reeves’s muntjac, ∼5.4 cm and 1-2 times) and to the antler-less small deer species such as the Chinese water deer (*Hydropotes inermis*). Given the range of phenotypes across the deer species and their common evolutionary origin from an antlered ancestor (Goss, 1983; Bubenik et al., 1990; Rössner et al., 2020), here, we selected four deer species (Cervinae subfamily: sika deer and muntjac; Capreolinae subfamily: reindeer and Chinese water deer) that span all two deer subfamilies in Cervidae (Heckeberg, 2020), namely, three-antlered deer (ATD) and the antler-less. We utilized both genome and AP transcriptome data (or the presumptive AP tissue in the case of Chinese water deer) of these four species to reveal antler-related genes through cross-species comparative analysis.

## Materials and Methods

### Sample Collection, RNA Preparation, and Sequencing

All AP tissues of the four deer species were collected from male deer and approved by the Animal Ethics Committee of the Institute of Antler Science and Product Technology, Changchun Sci-Tech University (CKARI202002). Sika deer and muntjac will start to initiate pedicle growth from the AP around the 6th month onward (approaching puberty), so AP was sampled from these two species at the age of 6 months. Chinese water deer do not grow antlers; thus, their AP-equivalent tissue can be sampled at any time. We collected AP-equivalent tissue from Chinese water deer at the age of 6 months in order to make the age of these species be consistent. Reindeer grow their pedicles at about 2 weeks of their age; thus, we sampled their AP tissues at the age of 2 weeks. A total of 12 pieces of AP tissues were collected (three biological replicates/species; four species), and these fresh tissues were immediately frozen in liquid nitrogen and then stored at −80°C for RNA extraction. Around 100 mg/tissue sample was rapidly ground into a fine powder using liquid nitrogen and a Freezer/Mill 6770 (SPEX CertiPrep Ltd., United States). Total RNA from each sample was isolated using a TRIzol reagent (Invitrogen Inc., Camarillo, CA) according to the manufacturer’s procedure. RNA quality was confirmed using an Agilent 2100 Bioanalyzer (Agilent Technologies Inc., United States), with a minimum RNA integrity number of 7.0. Six micrograms of total RNA were used to construct libraries according to the manufacturer’s instructions (Illumina TruSeq Library Preparation Kit v3). The library quality was assessed on the Agilent Bioanalyzer 2100 system, and molecular fragments with the length of 200–300 bp were selected and sequenced with 150 bp paired-end reads using an Illumina HiSeq 4000 instrument at Beijing Genomics Institute (Shenzhen, China). Information regarding the clean reads from the 12 samples that passed the quality filtering is summarized in Supplementary Table S1.

### Retrieval of the Currently Available Published Deer Genome Sequences

Genome sequences of four deer species were retrieved and downloaded from the published genome databases, including sika deer (CNGB: GWHANOY00000000), reindeer (GigaDB: DOI:10.5524/100370) (Li et al., 2017), muntjac (GenBank: GCA_008787405.2) (Mudd et al., 2020), and Chinese water deer (GenBank: GCA_006459105.1) (Chen et al., 2019). These genome sequences were of high-quality, such as both sika deer and muntjac have been assembled and annotated at the chromosome level.

### Obtaining Deer-Orthologs

In order to obtain high-quality deer-orthologs among four deer species, we developed a strict pipeline (Supplementary Figure S1) that combined their genome and transcriptome data to obtain protein-coding deer-orthologs based on the previous published methods (Fushan et al., 2015; Wang et al., 2016; Darbellay and Necsulea, 2020). First, we used Trinity v2.4.1 (Haas et al., 2013) to carry out *de novo* assembly from the quality-filtered reads for four deer species, respectively. The high-quality reads were also mapped on their corresponding reference genomes to generate reference-based transcripts by using HISAT2/StringTie (Pertea et al., 2016). Then, both the *de novo* and reference-based transcripts were merged using a CD-HIT-EST tool in CD-HIT Suite v3.0.3 (Li et al., 2006). Subsequently, the protein-coding sequences of the four species were predicted by TransDecoder v2.0.1 (Haas et al., 2013), respectively, and called transcriptome-based protein-coding datasets (Tset). We also derived the protein-coding sequences of the four species from their corresponding genomes based on gene annotation file, called genome-based protein-coding datasets (Gset). For the two datasets, we used cattle as the reference species to determine protein-coding orthologs, respectively, based on a best-bidirectional BLAST hit criterion (Overbeek et al., 1999). After joining these orthologs from both the Tset and Gset, we used InParanoid v4.1 (Ostlund et al., 2010) to obtain the deer-orthologs based on cattle orthologs (CT) as an outgroup. Finally, we obtained a total of 11,006 deer-orthologs among these four species.

### Principal Component Analysis and Hierarchical Cluster Analysis

To obtain the gene expression matrix (count and FPKM) of the 12 samples, the high-quality reads were mapped to their corresponding 11,006 deer-orthologs, respectively, with RSEM v1.3.0 (Li and Dewey, 2011). Both PCA and hierarchical cluster analyses were performed based on these 11,006 deer-orthologs by “prcomp” and “hclust” R function, respectively. Among these deer-orthologs, 9,540 (86.7%) had FPKM > 0.5 in all biological replicates of any one of the four species.

### Generation of Differentially Expressed Genes

Considering the different gene lengths between these deer-orthologs of the four species, the SCBN v1.10.0R package (Zhou et al., 2019) was used to search for the optimal scaling factors from the count matrix of the 11,006 deer-orthologs. The derived scaling factors were manually input to the DESeq2 v 2.1.18R package (Anders and Huber, 2010) to produce the DEGs. All *p* values were adjusted for multiple testing using the Benjamini–Hochberg method as implemented in DESeq2.

### Protein Sequence Divergence Analysis of Deer-Orthologs

The orthologous alignments between the ATDs and Chinese water deer were further conducted by using GBLOCKS (Castresana, 2000) to remove gaps and unreliable alignment columns. We then calculated the non-synonymous mutation rate (Ka) and the synonymous mutation rate (Ks) of these alignments by using the KaKs-calculator (Zhang et al., 2006) with the “MA” method. We excluded genes with Ks > 2 and Ka/Ks > 3 because high estimates of Ks may indicate saturation in synonymous sites or alignment errors (Uebbing et al., 2016).

### Construction of the Protein–Protein Interaction Network

The online database, STRINGdb (https://string-db.org/), was used to construct the protein–protein interaction (PPI) network, with all interaction sources and a minimum required interaction score being set at ≥ 0.4 for our genes. The Cytoscape v3.6 (Shannon et al., 2003) was used to visualize the protein–protein network. Network statistics were performed through in-house commands in the Cytoscape. Key hub nodes in the network were defined by their connective degrees with other nodes.

### Ingenuity Pathway Analysis

Functional enrichment of the data set was carried out using the IPA package (release date: 8 Feb 2019). Gene expression as defined by average fold change of all comparison groups in each gene set that we defined was used as the core analysis type with the Ingenuity Knowledge Base as the reference set. When analyses were performed, an adjust *p* value with the Benjamini–Hochberg method was set at < 0.01 and z-score (absolute value) > 2.

### Statistical Analysis

Correlation coefficients and the Wilcox test were performed using the “cor.test” and “wilcox.test” R functions, respectively. Significant differences (at least *p* value < 0.05) between two groups were determined.

## Results and Discussion

### Global Gene Expression Patterns of the AP Perfectly Match the Status of Antler Phenotype Variation

We analyzed gene expression data of the AP for 11,006 deer-orthologs to ascertain whether the global gene expression pattern matches the status of antler phenotypes across the four Cervidae species. Much of the variation in gene expression was evident in the comparison between ATDs and Chinese water deer, which separated on the first PCA axis (36.2% of the variation) (Figure 1B). The second PCA axis (26.7%) reflects the differences among the ATDs. This variation was also confirmed by a hierarchical cluster analysis among the four species (Figure 1C). The two large ATDs were closest to one another, followed by the small ATD (muntjac). The correlation of the ATDs and Chinese water deer was the lowest. These results showed that it is the global gene expression pattern in the AP tissues rather than the species evolutionary topology (Chen et al., 2019) that matches the status of antler phenotypes (Figure 1D), supporting the fact that the genes expressed in the AP may be related to antler phenotypes.

### Upregulated Genes in the ATDs Show Evidence of Co-Expression and Co-Variation

**FIGURE 2:**
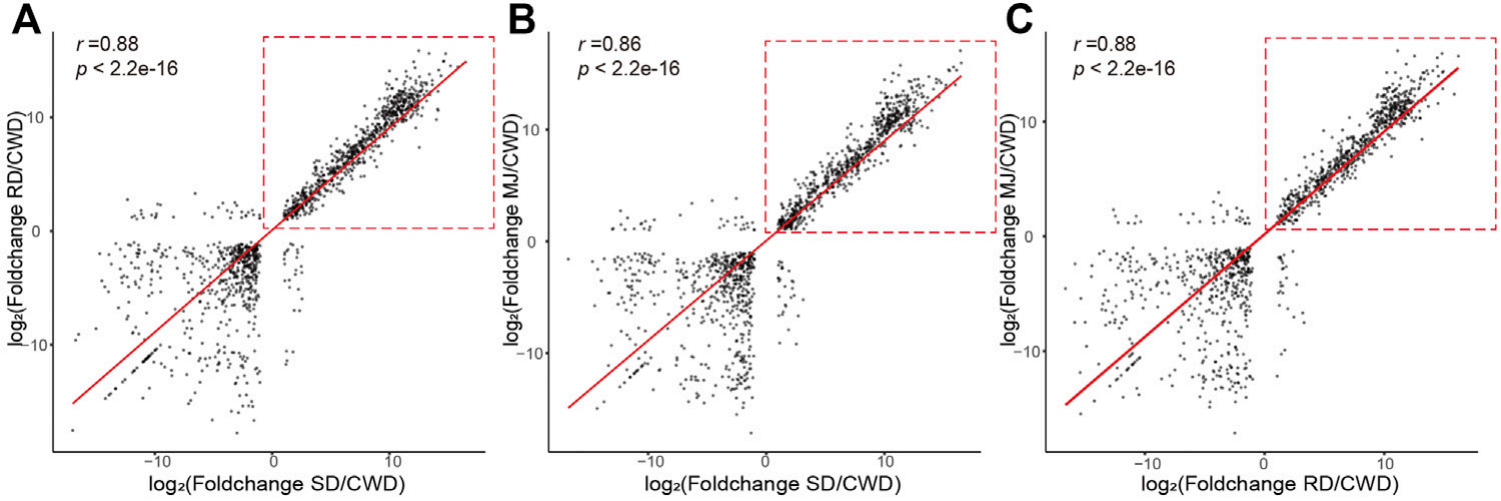
Correlation of the DEG sets (|log_2_FoldChange| ≥ 1 and adjusted *p* value < 0.01) of the ATDs relative to Chinese water deer: **(A)** SD/CWD vs RD/CWD, **(B)** SD/CWD vs MJ/CWD, and **(C)** RD/CWD vs MJ/CWD. Note that the high correlations were due mainly to the upregulated genes (red-dotted boxes). RD: reindeer, SD: sika deer, MJ: muntjac, and CWD: Chinese water deer.

First, we analyzed pair-wise correlation coefficients between the DEG sets (|log_2_FoldChange| ≥ 1 and adjust *p* value < 0.01; Supplementary Table S2). The results showed that the correlation coefficients (0.86–0.88) between three ATDs relative to Chinese water deer were higher than those of other groups (Figure 2), suggesting that these DEGs were likely involved in regulation of antler development. However, these high correlations were found to be mainly contributed by the upregulated genes (Figure 2, red dotted boxes).

**FIGURE 3:**
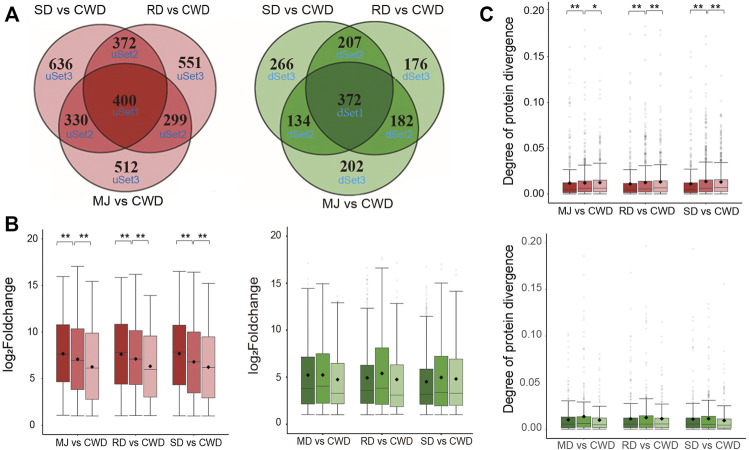
**(A)** Venn diagram of the upregulated (red) and downregulated (green) genes of three comparative groups respectively based on the overlap level. Note that the higher the overlap level, the higher co-expressed degree of the genes. **(B)** Boxplot of the changes in gene expression in the upregulated (red) and downregulated (green) gene sets of each of three comparative groups, respectively. Wilcox test **p < 0.01 and *p < 0.05. **(C)** Boxplot of the degree of protein divergence (non-synonymous mutation rate (Ka)) of deer-orthologs in the upregulated (red) and downregulated (green) gene sets of each of three comparative groups, respectively. Wilcox test **p < 0.01 and *p < 0.05.

Next, we asked whether these DEGs related to antler development among the ATDs would show evidence of co-expression. We divided the DEGs (|log_2_FoldChange| ≥ 1 and adjusted *p* value < 0.01) in each of the ATDs vs. Chinese water deer, respectively, into six gene sets based on the overlap level of these DEGs by applying Venn analysis (Figure 3A). Of these six gene sets, three were upregulated (uSet1 (400), uSet2 (1,001), and uSet3 (1,699)) and three downregulated (dSet1 (372), dSet2 (523), and dSet3 (644); Supplementary Table S3). Genes in Set1 were up/downregulated in all three comparative groups, those in Set2 were up/downregulated in any two of the three groups, and those in Set3 were up/downregulated in only one of the three groups. Expression changes of the upregulated genes in the uSet1, 2, and 3 of each comparative group decreased successively and significantly (Wilcox test, *p* value < 0.01; Figure 3B). In contrast, expression patterns in the downregulated genes did not show a similar trend. We further investigated divergence of their protein sequences among the DEGs in the six gene sets. We applied the Ka value (non-synonymous mutation rate) of protein orthologs to measure the degree of protein divergence. The results showed that the degree of protein divergence increased successively and significantly from uSet1 to uSet2 to uSet3 (Wilcox test, *p* value < 0.01) (Figure 3C). In contrast, there was no such trend detected for the downregulated genes. Taken together, these findings indicated the higher the co-expression degree of upregulated genes among the ATDs, the more conserved their protein sequences.

### Upregulated Genes in the ATDs Are Growth Related and Likely Participate in Antler Development

**FIGURE 4:**
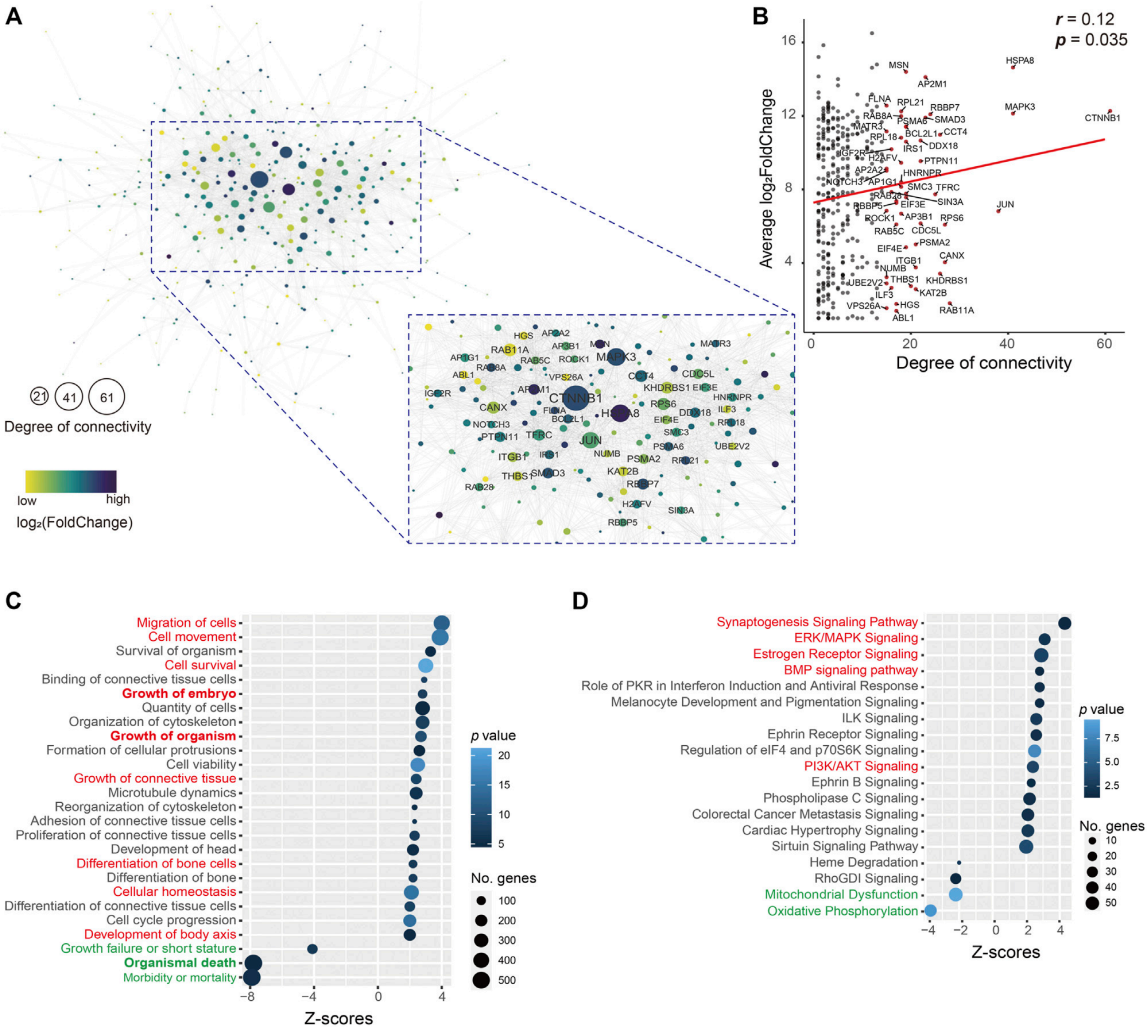
**(A)** Interactive network. This network consisted of 346 (86.5%) genes in the uSet. Average degree of connectivity was 7.4. Nodes with the degree of connectivity ≥ 15 are emphasized. Value of log_2_FoldChange of genes is indicated by the grade of the color. The size of the node indicates the degree of connectivity; the larger the node, the higher the degree of connectivity. **(B)** Correlation of 346 (86.5%) genes in the uSet1 between their degree and log_2_FoldChange. Gene names with the degree of connectivity > 15 are shown (red dot). Bubble plot of enrichment analysis results of **(C)** molecular/cellular functions and **(D)** canonical pathways in IPA software using all genes in the uSet1 and uSet2 and dSet1 and dSet2. The key upregulated (red) and downregulated (green) annotated terms are highlighted.

As these upregulated genes (400) in the uSet1 were the most co-expressed among the ATDs, these genes were used to construct the protein–protein interaction network using STRINGdb, and visualized by using Cytoscape. The network analysis results showed that the number of nodes was 346 (86.5%) and average degree of connectivity was 7.4 (Figure 4A), indicating this interaction network is robust. Furthermore, both the degree of connectivity and log_2_FoldChange of these genes was correlated (*p* = 0.035; Figure 4B), we focused on the role of key hub genes with the degree of connectivity ≥ 15 in the network, for example, *CTNNB1* (61), *MAPK3* (41), *JUN* (38), *ITGB1* (21), *THBS1* (20), and *BCL2L1* (19). The *CTNNB1* is a key downstream component of the canonical Wnt signaling pathway, which plays an important role in appendage regeneration (Stoick-Cooper et al., 2007; Ba et al., 2019). The protein THBS1 is reported to promote angiogenesis through interactions with a number of integrin heterodimers (e.g., ITGA3-ITGB1 complex) (Chandrasekaran et al., 2000). This is consistent with the *in vivo* findings that blood vessels are richly distributed in antler lineage tissues, even including antler cartilage, which is avascular in its somatic counterpart. The anti-apoptotic protein BCL2L1, upregulated by the MAPK/c-Jun signaling pathway (Zhang et al., 2018), is a potent inhibitor of cell death by blocking the voltage-dependent anion channel (VDAC) to prevent the release of death proteases called caspase (CASP) activator, cytochrome c (CYC) (Tsujimoto and Shimizu, 2000). The anti-apoptotic factors could be prerequisite for the formation of an antler tissue mass of 20 kg or so within 60 days from around 3 million cells (Li et al., 2009b).

We further utilized a total of 2,296 co-expressed genes in the uSet1, uSet2, dSet1, and dSet2 to perform an enrichment analysis of molecular/cellular functions and canonical pathways by applying IPA software. The analysis of molecular/cellular functions showed that of the enriched genes, those related to growth and development of the embryo/organism/body and differentiation of bone cells were highly upregulated (z-score > 2), while those related to organismal death and growth failure (morbidity and mortality) were highly downregulated (z-score < -2) in the ATDs as compared with the Chinese water deer, which are possibly related to the disappearance of antlers in the Chinese water deer during the course of evolution (Randi et al., 1998; Wang et al., 2019) (Figure 4C and Supplementary Table S4). These findings indicate that these co-expressed genes are involved in antler development and rescue antler failure.

The enrichment analysis of canonical pathways showed that the most upregulated signaling pathways were growth related (e.g., synaptogenesis, estrogen receptor, ERK/MAPK, BMP, and PI3K/AKT, z-score > 2) and the most downregulated signaling pathways included mitochondrial dysfunction and oxidative phosphorylation (z-score < -2; Figure 4D and Supplementary Table S5). Mitochondrial dysfunctions (e.g., *CASP9*, *CYC1*, *VDAC1*, and *VDAC2*) are a group of genetic disorders that are characterized by defects in oxidative phosphorylation (Gorman et al., 2016), which may be related to the disappearance of antlers in the Chinese water deer.

Among the upregulated pathways, synaptogenesis signaling was the most significantly enriched, which further supports the neural crest origin of the AP cells (Price et al., 2005; Landete-Castillejos et al., 2019). Antlers are organs of bone and directly formed from the proliferation and differentiation of AP cells (Goss and Powel, 1985; Li and Suttie, 2000). The BMP pathway is required for chondrogenesis/osteogenesis and thus is closely associated with the antler development. In the BMP pathway, genes for *BMP1/2/5*, *BMPR2*, and *CREB1* were upregulated (Supplementary Table S3). BMPs are involved in endochondral bone formation and embryogenesis (Sasano et al., 1993). These proteins transduce their signals through the formation of heteromeric complexes of BMPR1 and 2. It was reported that estrogen receptor mediates cell proliferation through the cAMP/PKA/CREB1 axis in murine bone marrow mesenchymal stem cells (Chuang et al., 2020).

The activated ERK/MAPK and PI3K/AKT pathways in the present study were also identified in our previous studies (Li et al., 2012; Li et al., 2018). These two pathways are important for maintaining rapid cell proliferation. In the PI3K/AKT signaling pathway, genes for *BCL2L1*, *CCND1*, *CTNNB1*, and *RAF1* were upregulated (Supplementary Table S3). The gene *CCND1* (encoding activity of cyclin-dependent kinase), a cell cycle-dependent factor, involved in cell cycle progression, may promote proliferation of the AP cells. Interestingly, it is reported that a newly identified binding motif of the androgen receptor evolved upstream of the *CCND1* gene, and may result in female reindeer antler growth (Lin et al., 2019). The CDKN1A protein inhibits the activity of CCND1; it was downregulated in the present study (Supplementary Table S3), which thus may increase cellular proliferation. Given that a limited number of AP cells (around 3.3 million cells) can generate 10 kg or more of antler tissue mass within 60 days (Li and Suttie, 2001), *CCND1* and *CDKN1A*, two highly expressed DEGs in the AP of ATDs, would be at least one of the factors contributing to this phenomenal growth rate.

Overall, this is the first study on the genes involved in antler development through cross-species comparative analysis based on their genome and AP transcriptome data in the Cervidae, and it further demonstrates that the co-expressed genes in antlered deer may regulate antler development.

## Data Availability

The datasets presented in this study can be found in online repositories. The names of the repository/repositories and accession number(s) can be found at: https://www.ncbi.nlm.nih.gov/, PRJNA768490.
