# Supplementary figures and images for: Cross-Species Analysis Reveals Co-Expressed Genes Regulating Antler Development in Cervidae
Source: Front Genet. 2022 May 18;13:878078. doi: 10.3389/fgene.2022.878078 (PMC9157503; doi:10.3389/fgene.2022.878078)

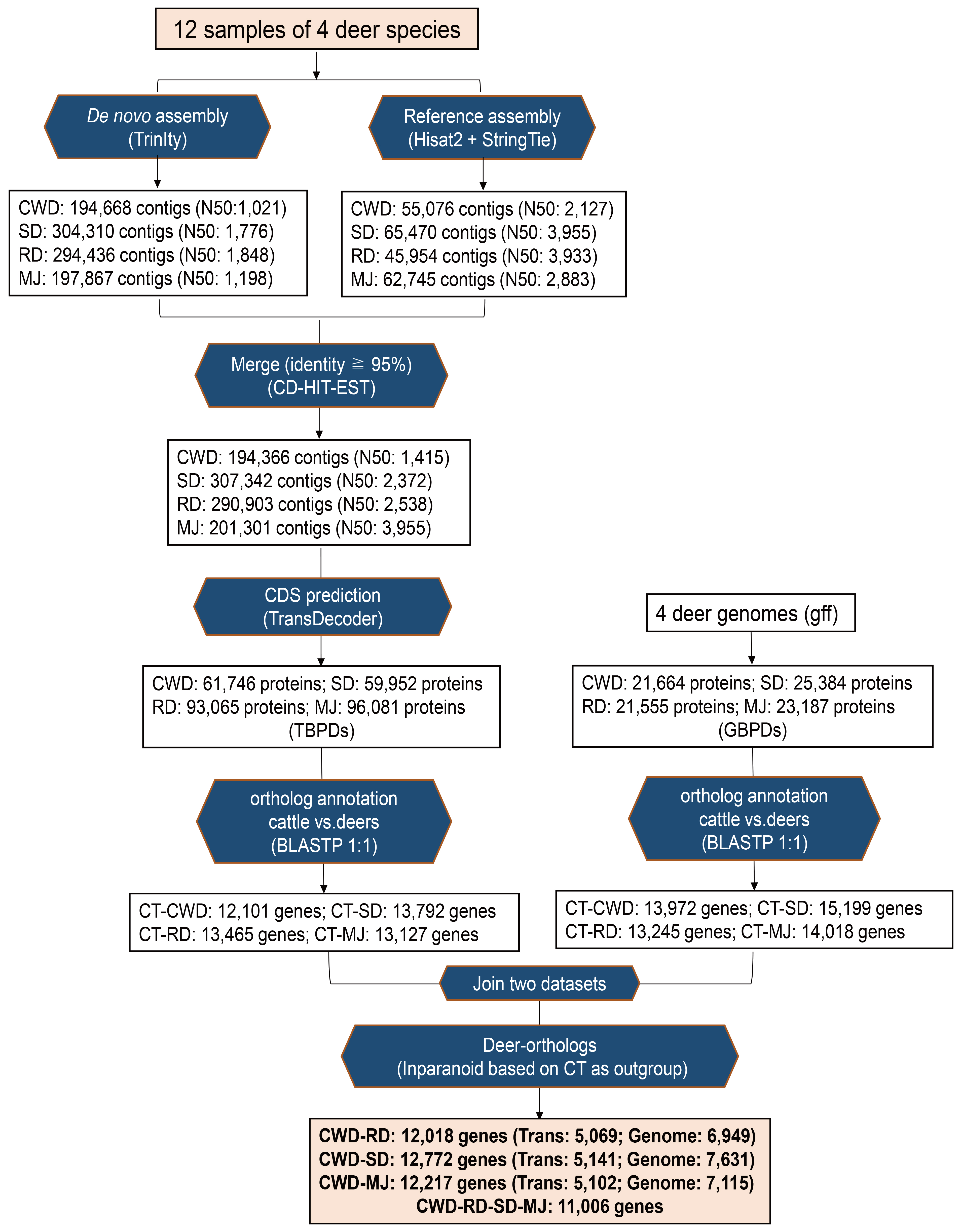

Supplement: Supplementary file 4 [file Image1.JPEG]
